# Efficacy and safety of esaxerenone (CS-3150) in primary hypertension: a meta-analysis

**DOI:** 10.1038/s41371-023-00889-9

**Published:** 2024-01-04

**Authors:** Ran Sun, Yali Li, Lei Lv, Weiliang Zhang, Xiaoxia Guo

**Affiliations:** Department of Metabolism, Shanxi Institute of Traditional Chinese Medicine, Taiyuan, 030000 China

**Keywords:** Hypertension, Cardiovascular diseases

## Abstract

This study aimed to assess the efficacy and safety of esaxerenone (CS-3150) in treating primary hypertension. PubMed (Medline), Cochrane Central Register of Controlled Trials (CENTRAL), and Embase databases were searched for articles published until April 18, 2023. The outcomes included were diastolic blood pressure (DBP), systolic blood pressure (SBP), 24 h DBP, 24 h SBP, and adverse events. The meta-analysis was conducted using RevMan 5.3. This study included three trials. CS-3150 5 mg had a greater effect on lowering the SBP, DBP, 24 h SBP, and 24 h DBP than either CS-3150 2.5 mg or eplerenone 50 mg. In contrast, CS-3150 2.5 mg and eplerenone 50 mg showed no significant difference in lowering DBP, SBP, 24 h DBP, and 24 h SBP. Moreover, adverse events occurred at comparable rates in the three groups. CS-3150 (especially CS-3150 5 mg) is an effective and safe treatment for primary hypertension; which can reduce blood pressure and alleviate hypertensive symptoms.

## Introduction

Hypertension is a major global health challenge and contributor to stroke, cardiovascular disease, and chronic kidney disease [1]. Globally, hypertension affects approximately one billion people, and this number is expected to increase to over 1,500,000 by 2025 [2, 3]. The proportion of individuals with hypertension varies worldwide, affecting 37.3% and 22.9% of individuals in developed and developing countries, respectively [4]. Hypertension can be categorized as secondary or primary based on its etiology. Primary hypertension, which has no identifiable secondary cause, is the predominant type of hypertension and affects more than 90% of hypertensive patients [5]. This makes it a major chronic, non-communicable disease globally.

Mineralocorticoid receptor (MR) antagonists are highly effective group of drugs in managing Primary hypertension [6]. A previous study showed that O-linked β-N-acetylglucosamine modification of MR enhanced its protein expression and transcriptional activity in vitro and in vivo under high-glucose conditions [7], suggesting a potential advantage of MR antagonists in patients with resistant hypertension and diabetic nephropathy. Currently, spironolactone, eplerenone, and esaxerenone (CS-3150) are clinically used as MR antagonists. Spironolactone binds poorly to MR and may induce adverse events, such as gynecomastia, menstrual irregularities, and impotence [8]. Eplerenone binds more specifically to MR than spironolactone; however, hyperkalemia remains a clinical issue [9, 10]. Esaxerenone, CS-3150, is a new nonsteroidal MR antagonist that has at least 1000 times higher selectivity for MR than other MR antagonists and does not exhibit antagonism for androgen, progesterone, and glucocorticoid receptors even at high doses [11]. CS-3150 potently blocked the binding of [3H]-aldosterone to MR, with a median inhibitory concentration (IC50) of 9.4 nmol/L, better than eplerenone and spironolactone (IC50 of 713 nmol/L and 36 nmol/L, respectively) [12]. Moreover, CS-3150 2.5 mg or 5 mg significantly and dose-dependently lowered sitting diastolic and systolic blood pressures (DBP and SBP) compared to the placebo [13]. These results suggested that CS-3150 is a highly selective and orally effective MR antagonist that can be used to manage renal disease, cardiovascular disease, and hypertension. This study comprehensively assessed the efficacy and safety of CS-3150 for the treatment of Primary hypertension through a meta-analysis to provide evidence-based support for its clinical management.

## Materials and methods

### Search strategy

PubMed (Medline), Cochrane Central Register of Controlled Trials (CENTRAL), and Embase databases were searched for studies published until April 18, 2023, with no language restrictions. The search terms used were hypertension, Primary hypertension, Esaxerenone, and CS-3150. In addition, we conducted a manual search of relevant domestic and international journals as well as the EU and Japanese clinical trial registry databases.

### Selection criteria

The inclusion criteria were: (1) people diagnosed with Primary hypertension (age ≥ 18), (2) studies with a randomized controlled trial (RCT) design, (3) the intervention group received CS-3150 and the comparison group received eplerenone or placebo, and (4) inclusion of at least one of the following outcome metrics: change from baseline in DBP, SBP, 24 h SBP, 24 h DBP, incidence of hyperkalemia, and any adverse events.

Exclusion criteria were as follows: (1) studies on secondary hypertension (2) non-RCT designs such as case reports, observational studies, reviews, conference abstracts, and protocols and (3) Incomplete data published.

### Data extraction

Two independent investigators extracted the following information: (1) study information, such as first author, publication year, sample locations, sample size, age, and sex; (2) type of therapy, dosage, SBP and DBP at baseline, 24 h SBP, 24 h DBP at baseline; and (3) primary and secondary outcomes. The risk of bias was assessed using the Cochrane Collaboration’s risk-of-bias tool. Any disagreements regarding study selection, data extraction, and risk of bias assessment were resolved by a third investigator.

### Statistical analysis

RevMan 5.3 was used to analyze the data. Continuous outcomes were reported as mean difference (MD) with a 95% confidence interval (CI), and categorical outcomes as relative risk (RR) with a 95% CI. Heterogeneity among the studies was assessed using the I^2^ value, where I^2^  >  50% indicated significant heterogeneity. A fixed-effect model or a random-effect model was applied depending on whether I^2^ ≤ 50% or not. To evaluate the robustness of the meta-analysis results, a sensitivity analysis was conducted.

## Results

### Characteristics of the included studies

**Fig. 1:**
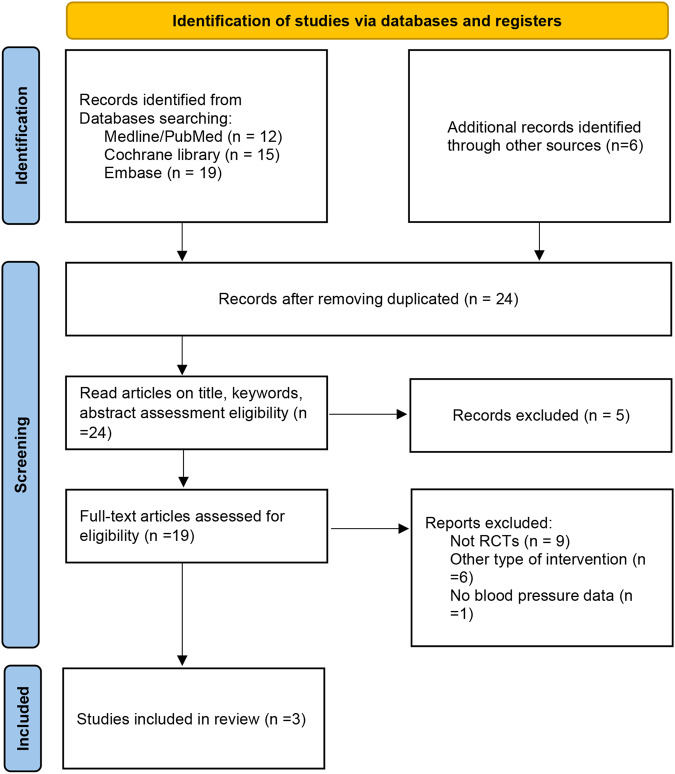
Flow diagram of the study selection.

Initially, 52 potentially relevant articles were identified, of which 28 were duplicates and were excluded. Of the remaining 24 articles, 5 were excluded after screening their titles and abstracts. Of the 19 articles, 16 were excluded after assessing their full texts for eligibility for various reasons, including other types of interventions (n = 6), not RCTs (n = 9), and no blood pressure data (n = 1). Finally, three RCTs were selected for quantitative analysis (Fig. 1).

The three RCTs included 1591 patients and were published between 2012 and 2020. The participants were aged between 20 and above, 11.4% of the which were over 65 years old, and had baseline blood pressure blood pressure levels of DBP ≥ 90–110 mmHg, SBP ≥ 140–180 mmHg, and 24 h SBP/DBP ≥ 130/80 mmHg (supplementary table 1).

### Quality assessment

**Fig. 2:**
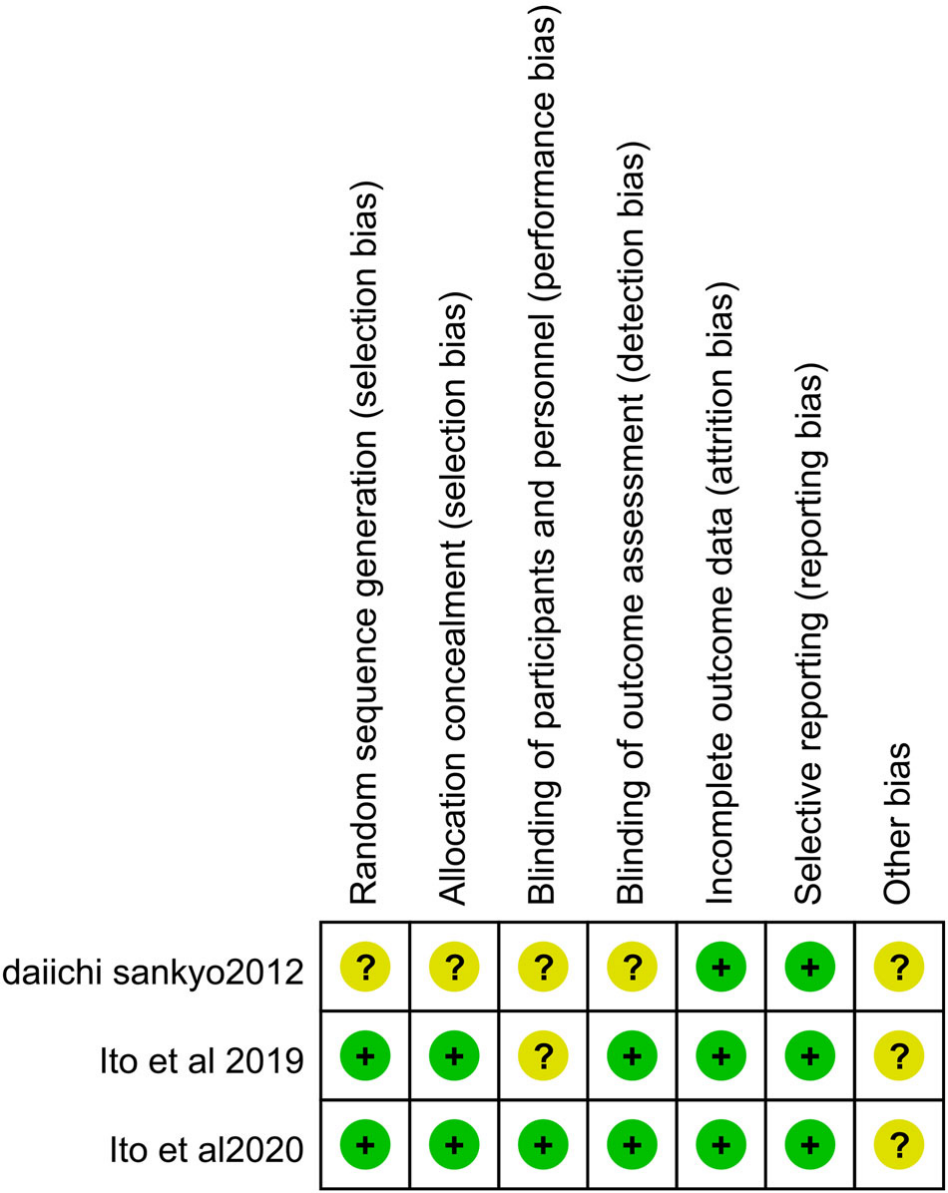
Risk of bias summary.

Figure 2 presents the risk of bias assessment of the included studies. Overall, the quality of the included literature was moderate. Most studies used a double-blind approach, allocation concealment, and random allocation, but did not specify the methods used. two of the three included studies did not perform a sample size calculation. The three studies had a short observation period, only about 2 weeks, so long-term observation indicators such as mortality were not evaluated. In one of the studies, the results of the experiment were disclosed in the JRCT database, and the exact execution of the experiment was obtained by contacting Daiichi, the esaxerenone experimental institution, by email, and the description of how it was randomly and how it was allocated was not sufficient. Other sources of bias were unclear for all RCTs.

### SBP

**Fig. 3:**
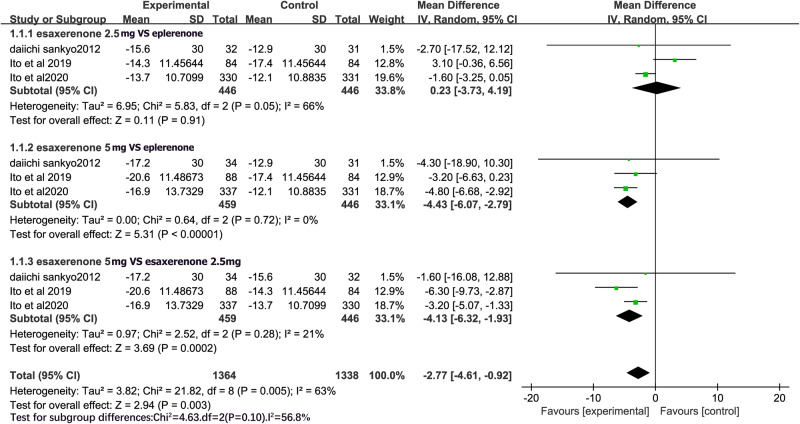
Forest plot of systolic blood pressure (SBP).

All RCTs compared the efficacy of eplerenone 50 mg and CS-3150 2.5 mg on SBP. Due to the significant heterogeneity among the studies (I^2^ = 66%), a random-effects model was chosen for the meta-analysis. The meta-analysis revealed that eplerenone 50 mg and CS-3150 2.5 mg showed no significant difference in SBP (MD = 0.23, 95% CI: −3.73, 4.19; P = 0.91). All RCTs compared the efficacy of CS-3150 5 mg with that of eplerenone 50 mg and CS-3150 2.5 mg on SBP. No heterogeneity was observed among the studies (I^2^ = 0% and I^2^ = 21%); therefore, a fixed-effects model was used. The meta-analysis found that CS-3150 5 mg reduced SBP more than eplerenone 50 mg (MD = −4.43, 95% CI: −6.07, −2.79; P < 0.00001) and CS-3150 2.5 mg (MD = −4.13, 95% CI: −6.32, −1.93; P = 0.0002) (Fig. 3).

### DBP

**Fig. 4:**
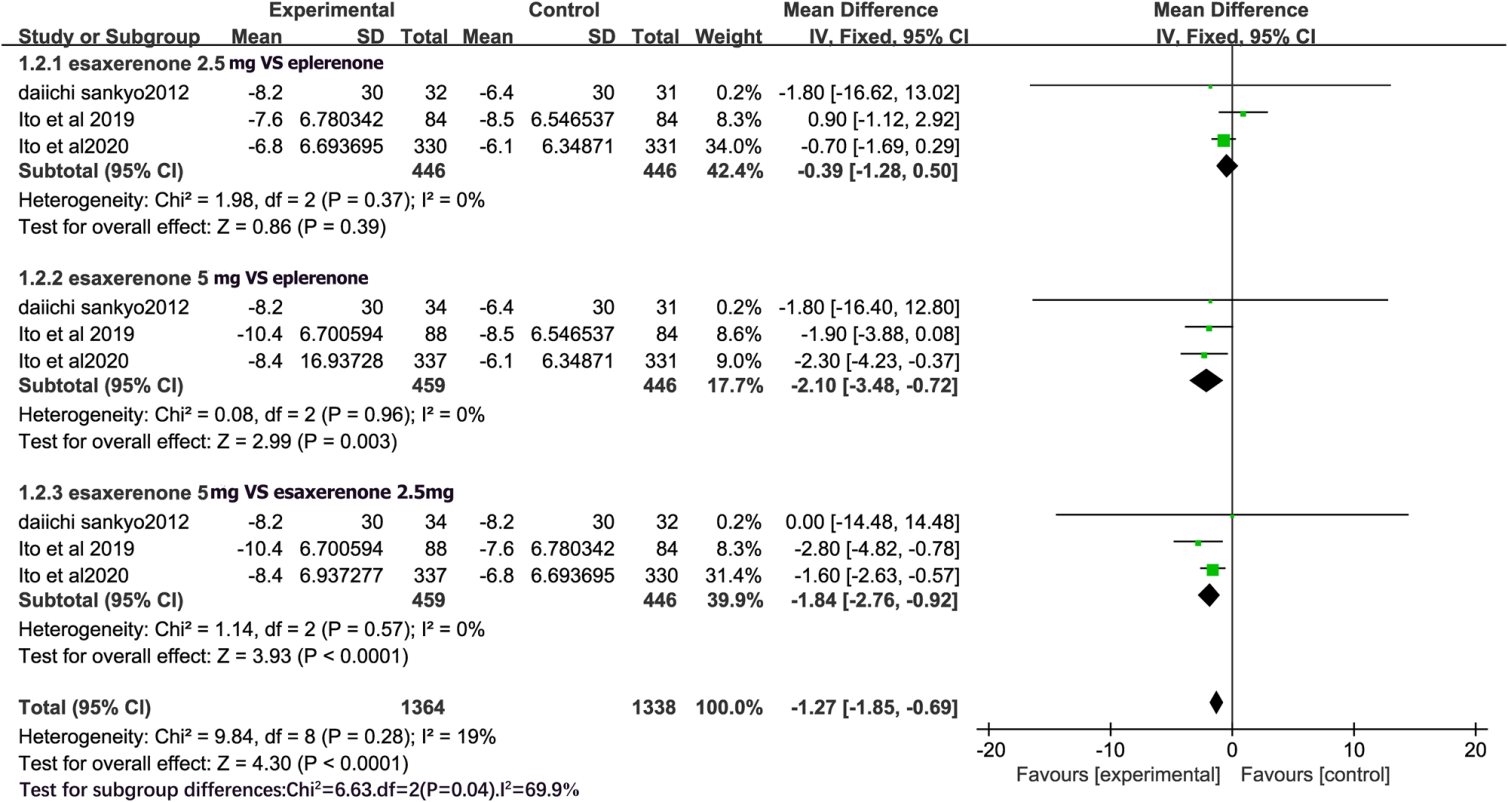
Forest plot of diastolic blood pressure (DBP).

All studies reported DBP. The studies were homogeneous (I^2^ = 0%), and fixed-effects models were used for the analysis. DBP did not significantly differ between CS-3150 2.5 mg and eplerenone 50 mg (MD = −0.39, 95% CI: −1.28, 0.50; P = 0.39). However, CS-3150 5 mg resulted in a significantly greater decrease in DBP compared with CS-3150 2.5 mg (MD = −1.84, 95% CI: −2.76, −0.92; P < 0.0001) and eplerenone 50 mg (MD = −2.10, 95% CI: −3.48, −0.72; P = 0.003) (Fig. 4).

### 24 h SBP

**Fig. 5:**
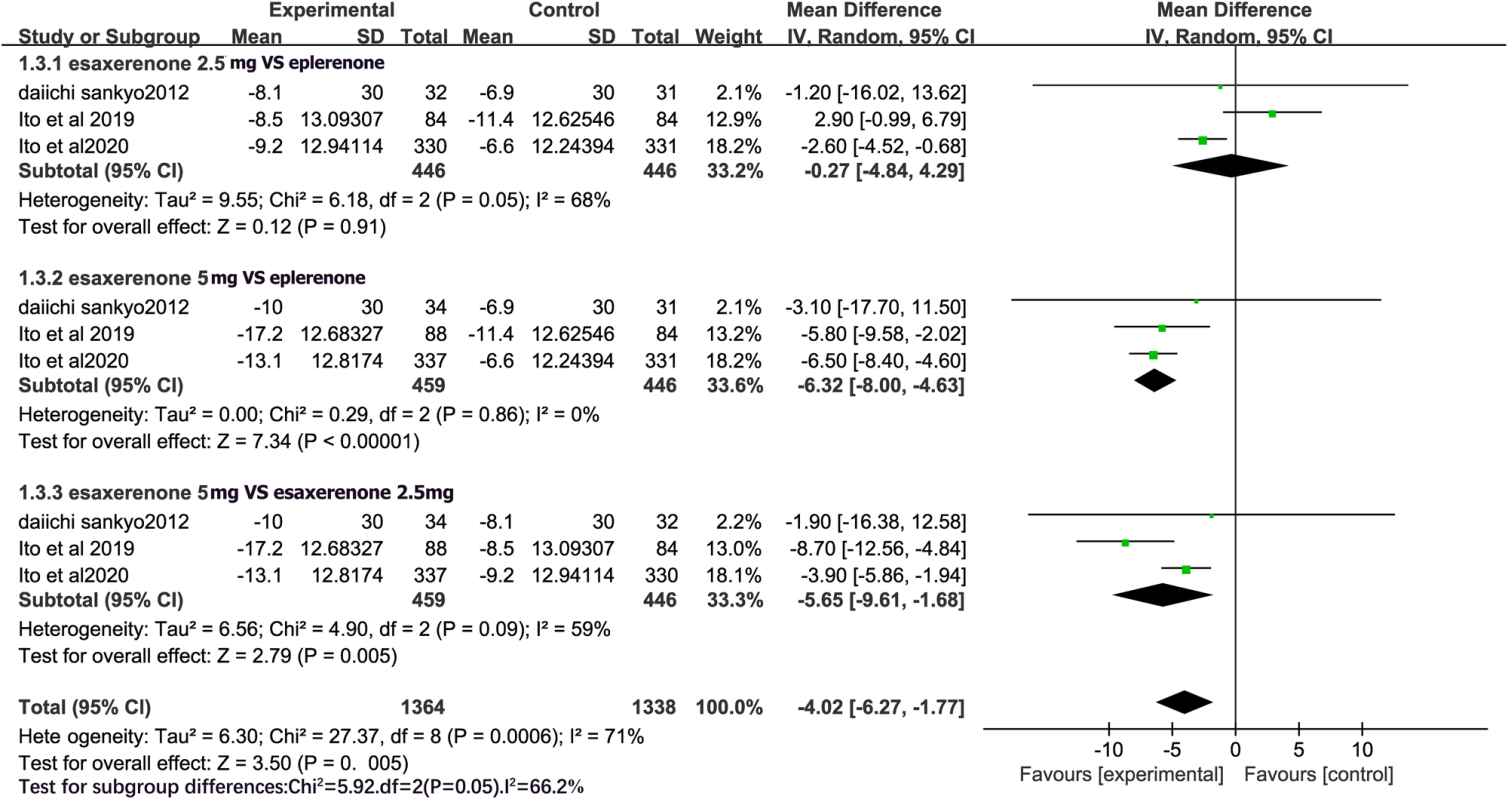
Forest plot of 24 h SBP.

Figure 5 shows the meta-analysis of 24 h SBP for all RCTs comparing CS-3150 2.5 mg with eplerenone 50 mg, and CS-3150 5 mg with eplerenone 50 mg and CS-3150 2.5 mg. A random-effects model was employed to account for the significant heterogeneity among the studies (I^2^ = 68% and I^2^ = 59%) when comparing CS-3150 2.5 mg with eplerenone 50 mg, and CS-3150 5 mg and CS-3150 2.5 mg on 24 h SBP. A fixed-effects model was employed to analyze the effects of eplerenone 50 mg and CS-3150 5 mg on 24 h SBP, as the studies were homogeneous (I^2^ = 0%). 24 h SBP did not significantly differ between CS-3150 2.5 mg and eplerenone 50 mg (MD = −0.27, 95% CI: −4.84, 4.29; P = 0.91). However, the result showed that CS-3150 5 mg could significantly lower 24 h SBP compared with CS-3150 2.5 mg (MD = -5.65, 95% CI: −9.61, −1.68; P = 0.005) and eplerenone 50 mg (MD = −6.32, 95% CI: −8.00, −4.63; P < 0.00001).

### 24 h DBP

**Fig. 6:**
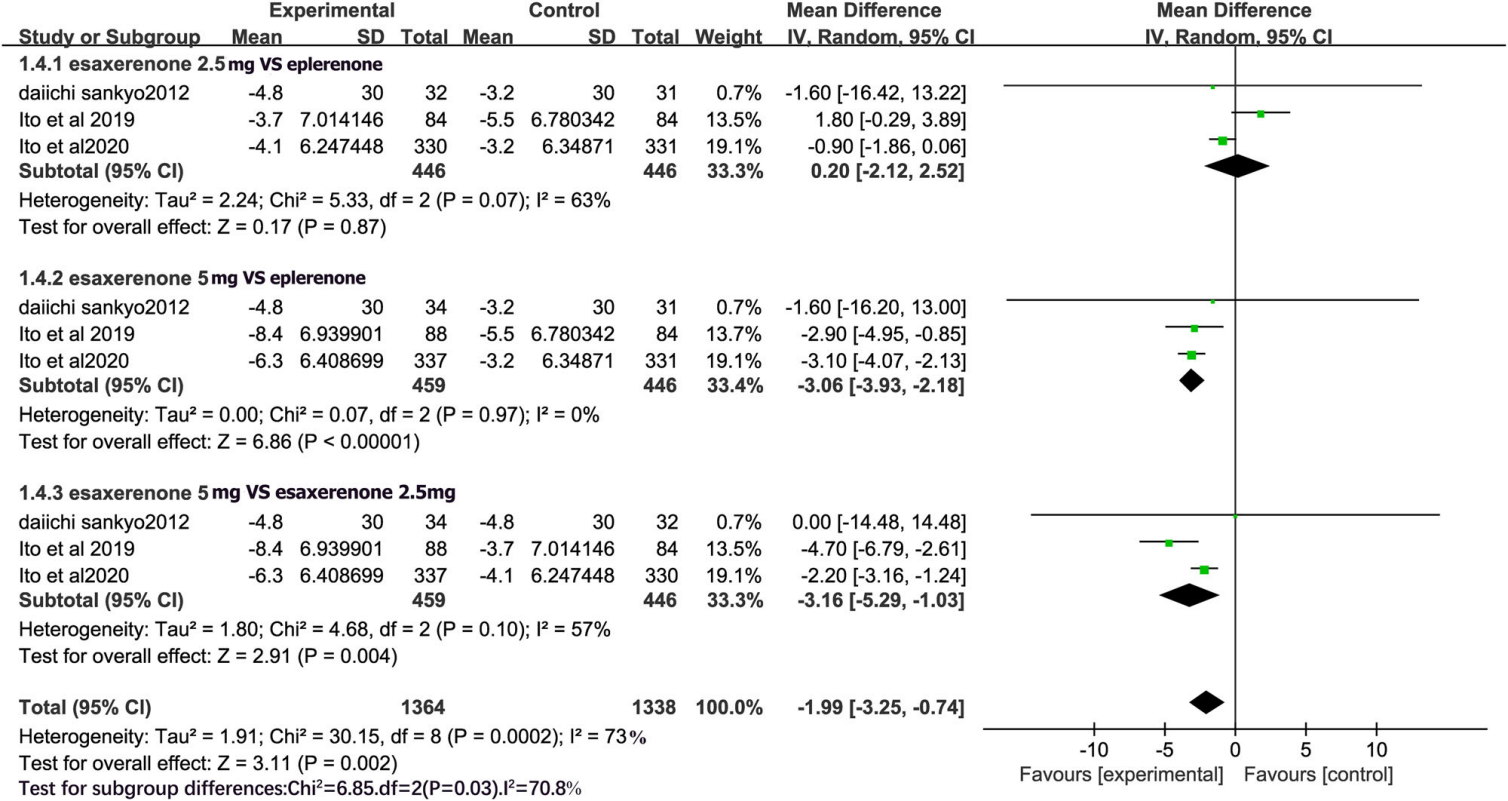
Forest plot of 24 h DBP.

All RCTs evaluated the efficacy of CS-3150 2.5 mg versus eplerenone 50 mg, and CS-3150 5 mg versus eplerenone 50 mg and CS-3150 2.5 mg on 24 h DBP. Because of the significant heterogeneity among the studies (I^2^ = 63% and I^2^ = 57%, respectively), a random-effects model was used to compare the effects of CS-3150 2.5 mg, eplerenone 50 mg, and CS-3150 5 mg on 24 h DBP; whereas a fixed-effects model was used to compare the effects of eplerenone 50 mg and CS-3150 5 mg on 24 h DBP (I^2^ = 0%). Compared to eplerenone 50 mg, CS-3150 2.5 mg had no significant effect on 24 h DBP (MD = 0.20, 95% CI: −2.12, 2.52; P = 0.87). However, CS-3150 5 mg significantly reduced 24 h DBP compared with both CS-3150 2.5 mg (MD = -3.16, 95% CI: −5.29, −1.03; P = 0.004) and eplerenone 50 mg (MD = −3.06, 95% CI: −3.93, −2.18; P < 0.00001) (Fig. 6).

### Adverse events

**Fig. 7:**
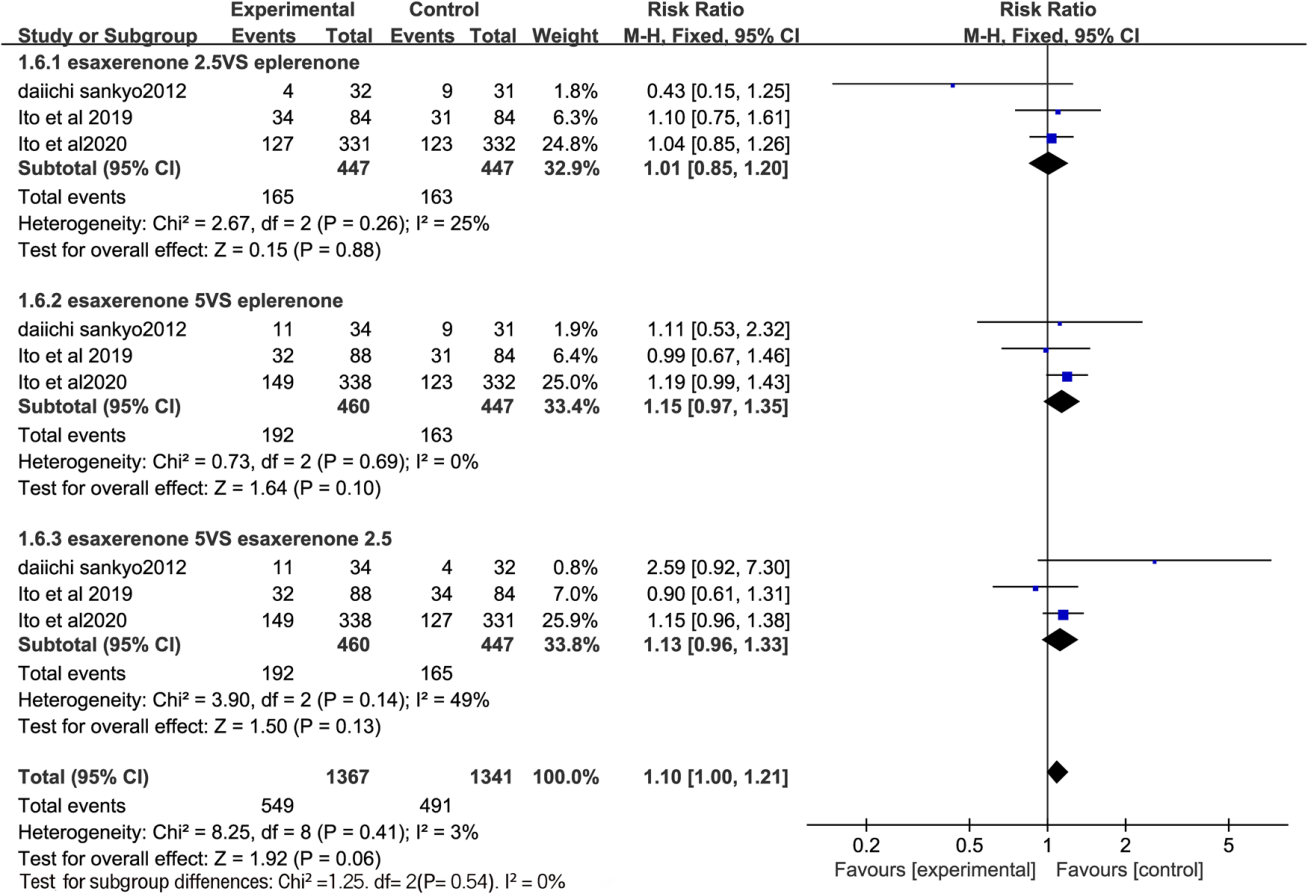
Forest plot of any adverse events.

All the RCTs reported adverse events. A fixed-effects model was employed for all comparisons, as there was no significant heterogeneity among the studies (I^2^ = 25%, I^2^ = 0%, and I^2^ = 49%, respectively). No statistical difference in any adverse events was found between CS-3150 2.5 mg and eplerenone 50 mg (RR = 1.01, 95% CI:0.85, 1.20; P = 0.88), CS-3150 5 mg and eplerenone 50 mg (RR = 1.15, 95% CI:0.97, 1.35; P = 0.10), and CS-3150 5 mg and CS-3150 2.5 mg (RR = 1.13, 95% CI:0.96, 1.33; P = 0.13) in terms of any adverse effects (Fig. 7).

**Fig. 8:**
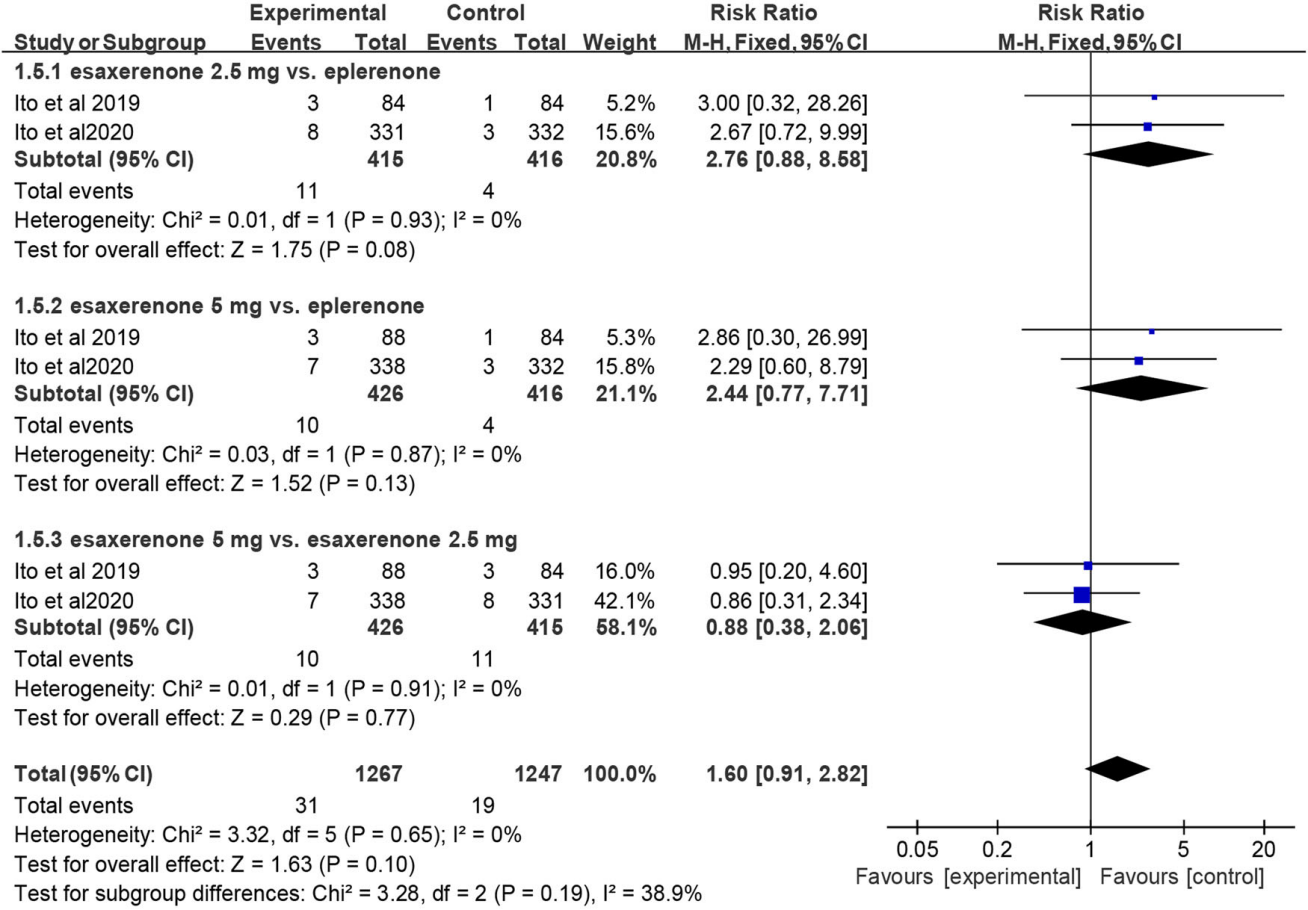
Forest plot of hyperkalemia.

Two RCTs reported hyperkalemia. Since the studies that assessed the outcomes of these interventions on hyperkalemia showed no significant heterogeneity (I^2^ = 0% for all comparisons), a fixed-effects model was applied. The meta-analysis indicated no statistically significant difference in the risk of hyperkalemia between CS-3150 2.5 mg/5 mg and eplerenone (RR = 2.76, 95% CI:0.88, 8.58; P = 0.08 for CS-3150 2.5 mg vs eplerenone; RR = 2.44, 95% CI:0.77, 7.71; P = 0.13 for CS-3150 5 mg vs eplerenone), or between CS-3150 2.5 mg and 5 mg (RR = 0.88, 95% CI:0.38, 2.06; P = 0.77 for CS-3150 5 mg vs CS-3150 2.5 mg) (Fig. 8).

### Sensitivity analysis

Sensitivity analyses were performed by omitting one study at a time to evaluate the reliability of the meta-analysis. These results were consistent and did not significantly change when a single study was omitted.

## Discussion

Early cardiovascular disease can be caused by Primary hypertension, which is a major modifiable risk factor for early death and disability around the world [14, 15]. According to global statistics, approximately 100 million people are affected by hypertension, which results to approximately 70,000 deaths annually [16].

Current first-line treatments for hypertension include diuretics, angiotensin-converting enzyme (ACE) inhibitors, and/or angiotensin receptor blockers (ARBs). For patients who do not respond to first-line treatment, combination treatment with two or three agents is recommended, and for those who still do not respond, treatment with mineralocorticoid receptor blockers (MRBs) are recommended. MRBs are currently considered a fourth-line treatment. A few years after discovery of aldosterone, its excess was associated with elevated blood pressure. The current reports of the correlation between renin-aldosterone system activity and primary hypertension are gradually increasing, and clinical and biochemical data indicate that there is a gray area between primary hypertension, and primary aldosteronism [17]. Therefore, antihypertensive therapy for MR can also be effective.

MR antagonists are a class of drugs that effectively lower blood pressure and slow the progression of kidney disease by inhibiting excessive activation of aldosterone on MR, thereby reducing oxidative stress, inflammation, fibrosis, and vascular remodeling [9]. The MR antagonists currently used in clinical practice are spironolactone and eplerenone, both of which belong to the steroid class of MR antagonists with certain efficacy and safety but also some limitations [18]. Spironolactone, although having a strong antagonistic effect on MR, also cross-reacts with other steroid receptors, causing adverse reactions, such as breast development and sexual dysfunction [19, 20]. Eplerenone was found to improve all-cause mortality and cardiovascular mortality in patients with heart failure [21]. Eplerenone has a relatively higher selectivity for MR, but its antagonistic effect on MR is weak and may still cause adverse reactions, such as hyperkalemia [22]. However, there is currently no available evidence to determine the effect of eplerenone on clinically meaningful outcomes such as mortality or morbidity in hypertensive patients [23]. A new non-steroidal MR antagonist, CS-3150, has been approved for the treatment of hypertension. Studies have found that CS-3150 has comparable safety to eplerenone, with no serious hyperkalemia or renal impairment [24, 25].

This study compared the anti-hypertensive effects of CS-3150 and eplerenone in patients with Primary hypertension. We found that CS-3150 5 mg significantly lowered SBP, DBP, 24 h SBP and DBP, whereas CS-3150 2.5 mg and eplerenone 50 mg showed no significant BP reduction. These results suggested that CS-3150 has a dose-dependent antihypertensive effect and is more effective than eplerenone. These results are consistent with those of previous clinical studies. A long-term phase 3 study with a multicenter, open-label design reported that CS-3150 2.5 mg/day or 5 mg/day for 12 or 28 weeks significantly decreased sitting SBP and DBP in patients with Primary hypertension [26]. A post-hoc analysis also demonstrated that CS-3150 improved night-time BP and N-terminal pro-B-type natriuretic peptide, which is a cardiovascular risk marker, based on different nocturnal BP dipping patterns (dippers, extreme dippers, non-dippers, and risers) [27].

Compared to steroid MR antagonists, CS-3150 has better safety and tolerability. Steroid MR antagonists, such as spironolactone and eplerenone, may cause adverse reactions such as hyperkalemia, breast development, and sexual dysfunction. CS-3150 did not cause these adverse reactions in clinical studies, nor did it cause serious hyperkalemia or renal impairment. In this study, we found no statistically significant differences between CS-3150 and eplerenone in terms of adverse events and hyperkalemia.

Some limitations of this study should be acknowledged before translating these results to clinical settings. First, owing to the recent launch of CS-3150 in Japan last 2019, few clinical trials were available, hence only three articles were included in this study. The limited number of literatures may have introduced selection bias and statistical error. Second, this study did not perform a subgroup analysis for different factors such as age, sex, baseline blood pressure, and comorbidities, which may have affected the efficacy and safety of CS-3150. Third, this meta-analysis analyzed adverse events as a safety outcome without separately assessing the incidence of hyperkalemia among the three groups (CS-3150 2.5 mg, CS-3150 5 mg, and eplerenone). Finally, all three RCTs included in this meta-analysis were conducted with the support of the same pharmaceutical company, which may have potential publication bias. Therefore, more large-sample, multicenter, long-term RCTs are needed to validate the benefits and indications of CS-3150 in patients with Primary hypertension. In addition, there are new aldosterone synthase inhibitors, Lorundrostat and Baxdrostat, which have potential advantages over spironolactone and eplerenone, but the difference in efficacy and safety between aldosterone synthase inhibitors and esaxerenone requires further research in the future [28, 29].

## Conclusion

In our study, CS-3150 (especially CS-3150 5 mg) was found to have a significant antihypertensive effect in lowering blood pressure in patients with Primary hypertension. To better assess the efficacy of CS-3150 in treating hypertension, larger high-quality RCTs are required.

## Summary

### What is known about topic

- Esaxerenone, CS-3150, is a new nonsteroidal mineralocorticoid receptor antagonist that has at least 1000 times higher selectivity for mineralocorticoid receptor than other mineralocorticoid receptor antagonists.
- CS-3150 is a highly selective and orally effective MR antagonist that can be used to manage renal disease, cardiovascular disease, and hypertension.

### What this study adds

- This study comprehensively assessed the efficacy and safety of CS-3150 for the treatment of primary hypertension through a meta-analysis to provide evidence-based support for its clinical management.
- CS-3150 5 mg had a greater effect on lowering the SBP, DBP, 24 h SBP, and 24 h DBP than either CS-3150 2.5 mg or eplerenone 50 mg. CS-3150 2.5 mg and eplerenone 50 mg showed no significant difference in lowering DBP, SBP, 24 h DBP, and 24 h SBP. Moreover, adverse events occurred at comparable rates in the three groups.

### Supplementary information

Table S1 Characteristics of the included studies in the meta-analysis.

## Data Availability

The data analyzed in study is available from the corresponding author upon reasonable request.
