# Supplementary material for: Efficacy and safety of esaxerenone (CS-3150) in primary hypertension: a meta-analysis
Source: J Hum Hypertens. 2024 Jan 4;38(2):102–9. doi: 10.1038/s41371-023-00889-9 (PMC10844087; doi:10.1038/s41371-023-00889-9)
Supplement: Supplementary file 1 — Table S1 Characteristics of the included studies in the meta-analysis. [file 41371_2023_889_MOESM1_ESM.docx]

Table 1 Characteristics of the included studies in the meta-analysis.

| Study | Location | Disease | Therapy | Number of patients | Mean age (Years) | Baseline systolic blood pressure | Baseline diastolic blood pressure | Therapy duration |
| --- | --- | --- | --- | --- | --- | --- | --- | --- |
| Ito et al 2019 | Japan | Primary hypertension | Esaxerenone 1.25mg/d | 82 | 57.2 ± 9.3 | 156.4 ± 9.1 | 97.2 ± 5.5 | 12W |
| NCT02345044 | |  | Esaxerenone 2.5 mg/d | 84 | 56.8 ± 9.4 | 156.4 ± 8.4 | 98.6 ± 5.6 | |
|  |  |  | Esaxerenone 5 mg/d | 88 | 57.1 ± 8.8 | 157.4 ± 9.0 | 97.2 ± 5.4 | |
|  |  |  | Eplerenone 50mg/d | 84 | 56.5 ± 10.0 | 157.9 ± 8.4 | 98.4 ± 5.3 | |
|  |  |  | Placebo | 85 | 57.3 ± 9.1 | 156.7 ± 9.0 | 96.8 ± 5.0 | |
| Ito et al 2020 | Japan | Primary hypertension | Esaxerenone 2.5 mg/d | 330 | 55.8±9.9 | 155.1±9.6 | 98.1±5.8 | 12W |
| NCT02890173 | |  | Esaxerenone 5 mg/d | 337 | 55.9±9.2 | 155.6±9.6 | 97.8±5.4 |  |
|  |  |  | Eplerenone 50mg/d | 331 | 54.8±9.7 | 155.1±9.6 | 98.3±5.6 |  |
| daiichi sankyo 2012 | Japan | Primary hypertension | Esaxerenone 1.25mg/d | 34 | 54.9±7.85 | mean-SBP154.1mmHg、mean-DBP 97mmHg Only significant differences in treatment background factors in the treatment group were reported, and no raw data were available | | 6W |
| JapicCTI-121921 | |  | Esaxerenone 2.5 mg/d | 32 | 57.3±7.99 |  |  |  |
|  |  |  | Esaxerenone 5 mg/d | 34 | 54.6±8.58 |  |  |  |
|  |  |  | Esaxerenone 10 mg/d | 33 | 55.7±7.86 |  |  |  |
|  |  |  | Eplerenone 50mg/d | 31 | 56.3±6.64 |  |  |  |
